# How Far Can Interventions to Increase Income Improve Adolescent Mental Health? Evidence From the UK Millennium Cohort Study and Next Steps

**DOI:** 10.1177/26320770231204993

**Published:** 2024-03-21

**Authors:** Aase Villadsen, Elliott Aidan Johnson, Richard Cookson, Matthew Thomas Johnson

**Affiliations:** 1 University College London, London, UK; 2 Northumbria University, Newcastle upon Tyne, UK; 3 University of York, York, North Yorkshire, UK

**Keywords:** health inequalities, youth mental health, Millennium Cohort Study, Next Steps, longitudinal analysis

## Abstract

UK adolescents and young adults are facing increasing rates of mental health problems and extremely difficult economic circumstances. There is strong evidence that interventions to increase income during adolescence can mitigate conditions such as anxiety and depression. However, policymakers lack quantified risk differences in the probability of mental illness between different income groups by which to establish the prospective scale of mitigation. Here, we estimate risk differences using two longitudinal cohort studies: Millennium Cohort Study (cohort members born between 2000 and 2002) and Next Steps (born 1989–1990). We quantify the association between income and risk of depression at age 14, serious mental illness at age 17, and non-psychotic mental illness at age 25. We also conduct sensitivity analysis using numerous other markers of socioeconomic (SES) status. We estimate that those living in the poorest quintile group of households have a greater probability of mental health problems than the richest at ages 14, 17, and 25. We find that other markers of SES status—subjective financial strain, cohort member assessed wealth relative to friends, occupational class, and education—display steeper associations with mental health symptoms relative to associations between mental health symptoms and income. Our findings are likely to be conservative underestimates since they are likely to be attenuated by well-known and large measurement errors in both mental health problems and living standards during adolescence.

A growing body of evidence indicating a crisis in the mental health of young people in the UK. In February 2022, there were 420,314 open referrals in England to child and adolescent mental health services (NHS Digital, 2022), a 54% increase since the same month in 2020 (NHS Digital, 2020). As part of a study covering England, Scotland, and Wales, Pitchforth et al. (2019) analyzed 18 cross-sectional surveys from the Health Survey for England covering the period 1995 to 2014 that included 92,422 4- to 24-year-olds. Among 16 to 24-year-olds, they found that the prevalence of longstanding mental health conditions increased from 0.6% to 5.9%. Increases in Scotland and Wales over different periods were also observed. They concluded that this was not merely due to increased diagnosis and reporting of mental health problems but represents a real and persistent long-term increase in mental ill-health. The causes of this long-term increase are poorly understood, but it is plausible that economic conditions play a role (Kim & Hagquist, 2018). Recent generations of young people have been subject to the global financial crisis of 2007–2008 with subsequent deep austerity policies (Stuckler et al., 2017), a global pandemic followed by price shocks and cost-of-living crises (Andersen & Reeves, 2022) exacerbated by the war in Ukraine (Francis-Devine et al., 2023), and a general chipping away at a sense of opportunity due to steep increases in housing costs (Anderson, 2022; Crisp & Powell, 2017), reductions in pension entitlements (Anderson, 2022), and increasing signs of the effects of climate change such as extreme weather events (Lawrance et al., 2022). Unlike previous generations, this generation is seriously questioning whether they are likely to lead healthier and wealthier lives than their parents (Clemence, 2023). However, a lack of understanding of the pathways exists from these events to health outcomes and of potential economic means of mitigating the growing adolescent mental health crisis.

Given estimated UK annual costs relating to mental health conditions of almost £118bn (McDaid & Park, 2022) and the UK Government’s focus on prevention (Department of Health and Social Care, 2018), interest in interventions that address social determinants of mental health is increasing. In this regard, some of the authors of this study have argued elsewhere (M. T. Johnson et al., 2021, 2022) that Universal Basic Income (UBI) can serve as a general upstream intervention, and the Welsh Government is currently conducting a pilot of basic income for young people who are turning 18 and leaving the care of a local authority (see Welsh Government [2022] for eligibility) as the basis for further investigation of the impact of the policy. However, considerable uncertainty remains about the potential magnitude and impact interventions that increase income might have on adolescent mental health conditions such as anxiety and depression.

This article analyzes data drawn from longitudinal cohort studies, namely the Millennium Cohort Study (MCS) and Next Steps (NS), to examine the direction and magnitude of the association between income and adolescent mental health problems and how this varies between early adolescence, mid-adolescence, and young adulthood and different measures of socioeconomic (SES) status. In their systematic review of experimental evidence from high-income countries, Cooper and Stewart (2021) find strong evidence that household income has a causal effect on mental health in childhood and adolescence, and that results from longitudinal fixed effect studies may underestimate the strength of impact. However, most of this evidence comes from the United States, and there are not yet any randomized controlled trials of cash transfers of a representative size, scale, and design in Britain. We begin by presenting existing evidence on the specific mental health challenges young people face before identifying theoretical pathways from social determinants to health outcomes.

## The Specific Mental Health Challenges Among Young People

Evidence from a range of sources has indicated a crisis of mental health problems among UK adolescents. Drawing on the Avon Longitudinal Study of Parents and Children (born 1991–1992) and MCS (born 2000–2002), Patalay and Gage (2019) found that 14-year-olds in 2015 were significantly more likely than those in 2005 to experience depressive symptoms (14.8% vs. 9%) and self-harm (14.4% vs. 11.8%). Indeed, the study reported a 60% increase in the rate of severe depressive symptoms. In addition to higher rates of depressive symptoms and self-harm, adolescents also report reduced average daily sleep duration and increased body mass index. By contrast, contemporary cohorts of adolescents report lower engagement in substance use such as cigarettes, alcohol, and cannabis relative to previous cohorts (Patalay & Gage 2019). Using data from two waves of the Longitudinal Study of Young People in England, Lessof et al. (2016) found that year 10s (14- to 15-year-olds) in 2014 were markedly more “work focused” than their counterparts in 2005 and that their mental well-being—particularly among girls—had worsened, with feelings of control over their own destinies reduced. Indeed, the Program for International Student Assessment 2018 results (OECD, 2019) found that UK 15-year-olds had the second lowest life satisfaction out of 30, above only Turkey, and had reported the greatest drop in life satisfaction between 2015 and 2018. It also found that UK 15-year-olds had some of the lowest levels of agreement among the 73 countries and regions surveyed with the following statements: “my life has clear meaning or purpose” (57%); “I have discovered a satisfactory meaning in life” (52%); and “I have a clear sense of what gives meaning to my life” (58%). Meanwhile, a US study of 1,500 respondents by Mind Share Partners (2019) found that 75% of Gen Z respondents (aged 16–22 in this study) had left roles for mental health reasons, both voluntarily and involuntarily, compared to 50% of Millennials (aged 23–38) and 34% of respondents overall. Meanwhile, Gen Z, Millennials, and Gen X (39–54) respondents were 4, 3.5, and 2 times, respectively, more likely to have been diagnosed with a mental health condition compared to baby boomers (55–73).

These trends are particularly concerning for public health in general and public budgets in particular, given that Solmi et al.’s (2022) meta-analysis of 192 epidemiological studies from around the world, including 708,561 participants, found that for any mental disorders, the proportion of individuals with onset before the ages of 14, 18, and 25 were 34.6%, 48.4%, and 62.5%, respectively. Mental health conditions at this young age are associated with “enduring disability, including school failure, impaired or unstable employment, and poor family and social functioning, leading to spirals of dysfunction and disadvantage that are difficult to reverse” (McGorry et al., 2007, p. S5). As such, it is crucial that means of mitigating social determinants are found to prevent life-long debilitating ill-health (E. A. Johnson et al., 2023).

### Work, Income, and the Social Determinants of Ill-Health

The increase in mental health problems among young people has occurred during a period of significant economic stress over the past 15 years. Twenty-six percent of working 18- to 29-year-olds said that they struggle to make ends meet financially, compared with 21% of respondents overall (Business in the Community, 2019). This has been compounded by the COVID-19 pandemic since those aged 25 and under are significantly more likely to have been employed in sectors that are at particular risk of job losses due to public health measures (TUC, 2020) and significantly more likely to have been made redundant consequently (Office for National Statistics, 2021, Table 28). This comes at a time during which the percentage of young people aged 21 to 24 living with parents has increased from 41.7% in 2006 (the same as 10 years previously) (Office for National Statistics, 2019) to 54.7% in 2021 (Office for National Statistics, 2022, Table 2021), delaying independent living and the health-promoting autonomy that brings (Patton et al., 2016). As such, inequality between generations and within the 14- to 24-year-old cohort is increasing. In sum, adolescents living in the UK have less money overall, less stable sources of money in particular, and possess fewer opportunities of earning a steady income through employment relative to previous cohorts.

Both cross-sectional and longitudinal associations between income and health (more broadly) are ubiquitous and have been established in a number of systematic reviews examining: population health (e.g., Kim, 2017; Kondo et al., 2009; McCartney et al., 2019; Ray & Linden, 2018; Reche et al., 2019); child health, well-being, and educational outcomes (Cooper & Stewart, 2021); child mental health (Pickett & Wilkinson, 2015); and adult mental health (Tibber et al., 2021). Supporting Pickett and Wilkinson’s causal review (2015), Adeline and Delattre’s (2017) analysis endorsed both the absolute income hypothesis (a positive and concave effect of income on health) and the income inequality hypothesis (that income inequalities affect the health and well-being of nearly all members of a society). As such, the evidence supports the notion that an increase in income is the “ultimate ‘multipurpose’ policy instrument” (Mayer, 1997, p. 145). However, Benzeval et al. (2014, p. 52) argued there is “less clarity regarding the particular role of income as a health determinant or the mechanisms by which income modification interventions might affect health.”

Some of the authors of the present study have argued that changes in income affect health by several key features. First, the size of the change affects the ability to satisfy needs (M. T. Johnson et al., 2019). Second, the degree of security of income affects stress associated with exposure to the threat of destitution (M. T. Johnson & Johnson, 2019). Third, the predictability of income impacts “extrinsic mortality cues” that affect people’s investment in health-affecting behavior, especially with regard to substance use and relationship formation (Pepper & Nettle, 2017).

Welfare systems are presented as means of mitigating these factors. However, conditional welfare systems like the UK’s Universal Credit—eligibility for which depends on being out of work, working but on a low income, or being unable to work due to a health condition (Government Digital Service, 2023)—are often associated with poor outcomes. While there are likely other risk factors involved, receipt of welfare in high-income countries is associated with worse health outcomes (Shahidi et al., 2019) and increased psychological distress prevalence (Wickham et al., 2020). Our model suggests several explanations: current welfare schemes are “insufficient to offset the negative health consequences of severe SES disadvantage” (Shahidi et al., 2019, p. 8); conditionality and assessment inflict stress (Dwyer et al., 2020) and create perverse incentives for health-diminishing behavior (M. T. Johnson et al., 2021); and focusing on the poorest fails to mitigate broader determinants that affect society as a whole (see Marmot et al., 2010). It is for such reasons that organizations, parties, and commentators have called for an evaluation of UBI as an alternative (The Lancet, 2020).

Some of the authors of this study have presented a theoretical model of impact for UBI that suggests that schemes that provide regular, uninterrupted access to cash support at minimum income standard levels can improve outcomes by reducing poverty, stress, and health-diminishing behavior (M. T. Johnson et al., 2021). However, the scale of impact is unclear with regard to differential effects at different levels of income and among adolescents and young adults in particular. To understand those effects in the absence of UK representative trials, this article analyzes large longitudinal cohort datasets to answer four related questions:

1. What is the magnitude of the relationship between income and adolescent mental health?
2. What is the direction of this relationship?
3. Does this relationship differ between different stages of adolescence, specifically between early adolescence (age 14), mid-adolescence (age 17), and early adulthood (age 25), and between children and their parents?
4. Do these relationships differ if we focus on subjective measures of SES status that indicate awareness of inequality, low income, and experience of “financial strain”?

We begin with an analysis of the MCS covering two waves when participants were aged 14 and 17.

## Study 1: MCS

### Method

#### Participants

The MCS follows an initial sample of over 19,000 children born in the United Kingdom between 2000 and 2002. The first survey was carried out when cohort members were 9 months old, with regular follow-ups at ages 3, 5, 7, 11, 14, and 17, which is the most recent survey. The MCS is highly multidisciplinary with a rich and diverse set of variables, including income and SES circumstances of parents and assessments of the mental health of both cohort members and parents. A total of 13,283 young people were included in this present analysis (see multiple imputation details further below). This provides examinations of the relationship between SES circumstances from 9 months to 14 years of age and cohort members’ mental health at ages 14 and 17.

#### Research Ethics

Ethical approval was granted for each sweep of the MCS prior to data collection. Data were downloaded from the UK Data Service and handled and processed according to the End User License Agreement.

### Measures

#### Mental Health of Cohort Members

We analyzed data using two measures of cohort-member mental health.

##### Short Moods and Feelings Questionnaire (SMFQ)

The first mental health measure is the self-reported SMFQ (Angold et al., 1995) score at age 14. It assesses depressive symptoms in children and consists of 13 items, with overall scores ranging between 0 and 26. A score of 12 or above is indicative of clinical levels of depressive symptoms.

##### Kessler 6 (K6)

The second mental health measure is the self-reported K6 (Kessler et al., 2003) score at age 17. K6 screens for severe mental illness using a 6-item scale and scores range between 0 and 24, with a score of 13 or above considered in clinical range.

#### Mental Health of Main Parents or Guardians

We also used two measures of mental health in the main parents or guardians of cohort members.

##### Kessler 6 (K6)

The first parent/guardian mental health measure is the self-reported K6 score when their child was aged 17.

##### Malaise Inventory

The second parent/guardian mental health measure is the nine-item Malaise Inventory score, which ranges from 0 to 9 (Rutter et al., 1970) and was self-reported in the initial survey when their child cohort member was aged 9 months.

#### Income and SES

##### Net Equivalized Household Income

Net household income was reported by the main parent or guardian in banded responses, using a card with weekly, monthly, and annual bands of take-home income after tax and other deductions, and with sources of income implicitly including state benefits. From these income bands, a continuous income measure was derived through imputation, which is further detailed in the data documentation (Centre for Longitudinal Studies, 2020). This was then equivalized using the OECD-modified equivalence scale to adjust for the size and composition of the household. See Centre for Longitudinal Studies (2020) for details on the measurement and derivation of the income measure. Average equivalized net household income from age 9 months to 14 years (spanning the first six survey sweeps) was transformed into quintile groups for use in the current analyses.

##### Parent or Guardian Financial Strain

Financial strain was reported by the main parent or guardian, who was asked how well they feel they are managing financially on a five-point scale: 1 = Living comfortably, 2 = Doing all right, 3 = Just about getting by, 4 = Finding it quite difficult, and 5 = Finding it very difficult. The measure was treated as continuous, and responses were added up across the first six survey sweeps from age 9 months to 14 years. The overall measure was transformed into both deciles and quantiles for use in this study.

##### Cohort Member Assessed Relative Family Wealth

Cohort member assessment of their family’s economic situation was carried out at age 11 in a single question: “Compared to your friends, is your family richer, poorer, or about the same?”

##### Highest in Household Occupational Classification (NS-SEC)

Highest in household occupational classification was derived based on a range of questions relating to their employment circumstances using the five-level National Statistical Socio-economic classification (NS-SEC) (Office for National Statistics, 2016): (a) managerial and professional, (b) intermediate, (c) small employer and self-employed, (d) lower supervisory and technical, and (e) semi-routine and routine. This was assessed at 9 months or 3 years if missing. Classification for those who were unclassifiable were imputed as part of the multiple imputation process (see “Analysis” section below).

##### Highest in Household Education

Highest in household educational level was ascertained in the initial survey at 9 months or, if missing in the second survey, at 3 years old. The classification used was the National Vocational Qualification system (NVQ), ranging from NVQ1 (GCSEs or equivalent, grade C or below) to NVQ5 (a postgraduate degree), with an additional category for those with no educational qualifications. Those educated abroad were treated as missing cases and imputed as part of the multiple imputation process.

##### Socioeconomic Deprivation Indices

Two indices of SES deprivation were created based on the measures outlined above. These totaled the number of disadvantages experienced throughout childhood. The first consisted of three elements of disadvantage (Index 3): lowest income decile, highest financial strain decile, and cohort members reporting their family being poorer. The second index consisted of five elements (Index 5), adding to the first index: highest in household NS-SEC 5 (routine and semi-routine), and highest in household NVQ1 or no educational qualifications. Data from the MCS are available from the UK Data Service (University of London, Institute of Education, Centre for Longitudinal Studies, 2022).

#### Data Analysis

Multiple imputation was used in the current analyses to deal with missing data due to attrition from the study. This ensures more accurate population estimates than using the available data alone (Mostafa et al., 2021). At age 17, data were provided on a total of 10,757 cohort members out of the 19,519 children initially recruited for the study. Using chained equations, a sequence of univariate imputation methods with fully conditional specification of prediction equations, 50 datasets were generated using multiple imputations, restoring the MCS sample to age 11, which is within the recommended threshold of imputing no more than 50% of missing variables (Mishra & Khare, 2014). Others have demonstrated that it is appropriate to impute missingness at an even higher level if using a large sample size and with the use of appropriate auxiliary variables (Madley-Dowd et al., 2019). A large number of auxiliary variables were used in this study, which maximizes the plausibility of the “missing at random” assumption and improves the accuracy of imputed values and estimates in cohort studies (Mostafa et al., 2021). These included all available measures through childhood and adolescence of child mental health, child physical health, parent or guardian mental health, housing tenure, single parent or guardian status, and also Index of Multiple Deprivation from age 9 months to age 5 years and, at age 17, cohort members’ self-reports of physical health, obesity, and academic achievement (Ministry of Housing, Communities & Local Government, 2020). The final analytical sample consisted of 13,283 cohort members. Attrition between the initial survey and age 11 was adjusted for in subsequent analyses using weights created for this purpose. Weights were also adjusted for the complex sampling design of the MCS. For the substantial analyses examining the association between childhood SES circumstances and mental health, the prevalence of both cohort members and parent or guardian mental health measures was examined by each of the SES variables. No other variables (covariates) were included in the analyses. Comparisons between the highest income and SES group and less advantaged groups were carried out using logistic regression (due to the dependent mental health variable being binary), which provided odds ratios that signify the magnitude of mental health inequalities. Observations were independent as twins and triplets have been excluded from the study, and no risk of multicollinearity existed as no control variables were used.

Additional analyses examined whether the relationship between household income and mental health was similar for male and female cohort members. Because the main parents or guardians were predominately female, this could not be replicated for parent or guardian mental health. Instead, additional analyses for income inequalities in parent or guardian mental health involved controlling for parent or guardian mental health measured prior to income measurements, which mitigates against reverse causality as it is plausible that mental health affects household income.

All analyses, including imputations, were carried out in Stata version 16 (StataCorp, 2019).

### Results

The analytical sample, detailed in Supplemental Table 1, demonstrated diversity in key demographic features, including with regard to ethnicity, income, and SES status, as well as mental health outcomes.

#### SMFQ at Age 14

We began by examining the prevalence of clinical levels (score ≥12) of depressive symptoms of cohort members at age 14, as measured by the SMFQ, household income, and other SES measures. As shown in Supplemental Table 2, across the whole of the sample, the prevalence was 14.9%, with much higher rates in female (22.0%) than in male (8.3%) participants. In terms of household income and SES measures, the general pattern shows that cohort members from the most disadvantaged households had a higher prevalence of clinical-level depressive symptoms. However, the relationship was not completely monotonic for all measures. For example, rates of depressive symptoms were the highest in the second (17.7%) income quintile rather than in the lowest (15.4%). Inequalities in mental health between the most disadvantaged and most advantaged were strongest for parent or guardian financial strain (*OR* = 1.7, *p* < .001) and for cohort member assessed relative family wealth (*OR* = 2.1, *p* < .001). There was a weaker relationship between cohort member mental health and the highest in household occupational classification, and for the highest in household education the association appeared non-monotonic. This was also reflected in analyses of the indices where the mental health inequalities were greatest on Index 3, which focuses on income-related variables and, unlike Index 5, excludes education and social class. Having two or three income-related disadvantages (Index 3) was related to a substantial increase in clinical levels of depression, compared to those with no such disadvantage (28.9% vs. 14.0%; *OR* = 2.5, *p* < .001).

#### Kessler 6 at Age 17

These patterns were consistent in the following wave, at age 17, using a related but different measure of mood-related mental health problems (Kessler 6). Supplemental Table 3 shows the prevalence of clinical levels (≥13) of psychological distress among cohort members at age 17, as measured by the Kessler 6 (K6) questionnaire, by household income, and by other SES measures. Overall, the prevalence was similar at around 15.0%, and as before with around twice the rate in female (21.2%) than in male (9.2%) participants. In relation to income and other SES measures, the prevalence tended to be lowest in the most advantaged groups and highest in the most disadvantaged. At age 14, the highest rates of psychological distress were in those from the second worst-off income quintile. For other SES measures, the pattern at age 17 also mirrored that at age 14: the inequalities in psychological distress were largest for parent or guardian financial strain (*OR* = 2.0, *p* < .001) and for cohort member assessed family financial situation (*OR* = 2.2). Differences between the highest and lowest occupational classification were more modest but significant (*OR* = 1.3 *p* < .05), and for education we see a non-monotonic pattern with only borderline significant differences. Having multiple problems of income-related disadvantages (Index 3) was related to a substantial increase in clinical levels of psychological distress, compared to those with no such disadvantage (*OR* = 2.6, *p* < .001).

#### Main Parent or Guardian Kessler 6 Clinical Levels at Cohort Member Aged 17

We then compared data on Kessler 6 as reported by the parent or guardian of cohort members to see whether the nature of the relationships between income and mental health changes with age, as adolescents develop into adults and gain a degree of economic independence. A limitation of this approach, however, is that parents or guardians are, of course, from different generations than MCS cohort members, and a trend of increasing reporting of mental health problems among younger generations exists. The prevalence of clinical levels of psychological distress in the main parent or guardian (95% female) is shown in supplemental Table 4, assessed when the cohort members were aged 17. The overall prevalence was 6.6%, less than half the level of cohort members. However, the social gradients in mental health were considerably steeper than in the adolescent cohort members and exhibited a fully monotonic pattern in the gradient in the most disadvantaged group. In relation to income and other SES measures, psychological distress was much more prevalent in disadvantaged groups. The rate in the lowest income quintile compared to the highest income quintile showed an odds ratio difference of 9.8. Those managing the least well financially had 17.2 times the odds (*p* < .001) of having clinical levels of psychological distress than those who managed the best financially. Differences were also sizeable between educational groups; those with no formal qualifications had 10.8 times the odds (*p* < .001) compared to those with the highest level of education. Between NS-SEC groups, those in semi-routine and routine occupations had 3.8 times the odds (*p* < .001) of clinical levels of distress compared to those in managerial and professional groups. Overall, the prevalence was much higher in those experiencing multiple types of SES disadvantage (Index 3 and Index 5) compared to those without any disadvantage.

Although income and other SES variables were measured prior to the measurement of parent or guardian mental health, which mitigates against reverse causation, nevertheless a possibility exists that parent or guardian mental health has an effect on parent or guardian income rather than the other way around. To further examine this, we conducted analyses where prior parent or guardian mental health measured at the initial sweep (child cohort member aged 9 months) was included as a control variable. Results are shown in Supplemental Table 5 (Model 2). Prior parent or guardian mental health is significantly associated with later parent or guardian mental health. Specifically, for every standard deviation increase in psychological distress at sweep 1 (cohort members aged 9 months), there was a 1.67 *OR* increase in clinical levels of psychological distress at sweep 7 (cohort members aged 17). However, the association between income and parent or guardian mental health remains substantial and highly significant, suggesting that, over time, low income can have substantial cumulative effects on parent or guardian mental health.

## Study 2: NS

The analysis of the MCS established that significant associations exist between average equivalized household income from 9 months to 14 years and clinical levels of K6 and SMFQ measures of psychological distress at ages 14 and 17. It was also clear that the relationship between income and K6 was steeper and more clearly monotonic among the parents or guardians of cohort members than the cohort members themselves. Equally, the relationship between parent- or guardian-reported financial strain and cohort-member psychological distress was steeper and more monotonic than objective measures of income, social class, and educational attainment.

The latest available MCS data covered cohort members at age 17. To investigate whether older members of the cohort of interest (14- to 24-year-olds) more closely resembled 17-year-olds or their parents or guardians in MCS, we decided to analyze data from the NS cohort members at age 25.

Data from NS are available from the UK Data Service (University College London, UCL Institute of Education, Centre for Longitudinal Studies, 2022).

### Methods

#### Participants

NS commenced in 2004 and recruited a cohort of nearly 16,000 Year 9 (age 14) students from state and independent schools as well as pupil referral units in England. The study was set up with a focus on young people’s journeys from school to university, training, and entry into the labor market. However, other aspects of cohort members’ lives have been assessed, including mental health. Cohort members were followed up annually until age 20 and then at age 25, which is the most recent survey sweep and the data used in the current analysis involving 6,814 cohort members. Excluded from the sample were those who were still in training or education (around 6%), as the focus of the analyses were on cohort member’s own income and SES circumstances.

#### Research Ethics

Ethical approval was granted for each sweep of the NS prior to data collection. Data were downloaded from the UK Data Service and handled and processed according to the End User License Agreement.

### Measures

#### Mental Health

##### General Health Questionnaire (GHQ-12)

Cohort members reported their mental health at age 25 using GHQ-12 (Goldberg & Williams, 1988), designed to detect general (non-psychotic) mental health problems among primary care patients. The scale ranges from 0 to 12, with a clinical threshold of four and above.

#### Income and SES

All measures of income and other SES circumstances were taken when NS cohort members were aged 25.

##### Individual Income

Individual income was collected using a banded question, and from these, a weekly continuous measure of income was imputed using a range of predictor variables (Calderwood et al., 2021).

##### Financial Strain

Financial strain was assessed using the same measure as in the MCS, asking cohort members how they were managing financially. However, only one time point measurement was used in NS and the last two response options were combined because of small numbers: 1 = Living comfortably, 2 = Doing alright, 3 = Just about getting by, and 4 = Finding it quite or very difficult.

##### Occupational Classification (NS-SEC)

Cohort members’ occupational classification was also measured using NS-SEC, as in the MCS: (a) managerial and professional, (b) intermediate, (c) small employer and self-employed, (d) lower supervisory and technical, and (e) semi-routine and routine. It should be noted that the imputation of missing cases is not used in the current analyses of the NS.

##### Education

Cohort members’ education was measured using the NVQ classifications: ranging from NVQ1 (GCSEs or equivalent, graded C or below) to NVQ5 (a postgraduate degree) and with an additional category for those with no educational qualifications.

##### Socioeconomic Deprivation Indices

Two indices of SES deprivation were created. These added up the number of SES disadvantages experienced at age 25. The first consisted of two elements of disadvantage (Index A): being in the lowest income decile and finding it quite or very difficult to manage financially. The second index (Index B) added two further elements: having an NVQ1 or no educational qualifications and NS-SEC 5 (routine and semi-routine) or unclassifiable (i.e., those who never worked or long-term unemployed).

#### Data Analysis

The prevalence of cohort members’ clinical levels of mental health disorders was examined by each of the SES measures outlined above. No other variables (covariates) were included in the analyses. Comparisons between the best-off income and SES group and less advantaged groups were carried out using logistic regression (due to the dependent mental health variable being binary), which provided odds ratios that signify the magnitude of mental health inequalities. Observations were independent with no twins or triplets in this sample, and there is no risk of multicollinearity as no control variables were used. All analyses use weights developed specifically to adjust for survey design and attrition between the initial survey and the eighth wave at age 25.

Additional analyses were carried out to examine whether any factors or circumstances moderated the relationship between SES measures (here limited to income and financial strain) and mental health disorders. Potential moderators were sex assigned at birth, existence of children, whether living with parents or guardians or with a partner, housing tenure (whether their home is owned outright/with a mortgage or rented/other), and whether in school. Separate analyses were carried out for each level of the moderator, and then a model with an interaction between income and moderator and financial strain and moderator, respectively, was run to test for statistical significance of the moderator.

#### Limitations

Analysis of NS was undertaken only on one wave of data when cohort members were aged 25. The initial aim was to understand, indicatively, whether associations between SES and mental health at age 25—when more economic independence might be expected to be observed—would more closely reflect those of 14- and 17-year-old cohort members or their parents or guardians in the MCS data. This was an important check given an interest in a 14 to 24 age cohort and the effects that might be observed at transition points above school age. However, the cross-sectional method used means that we cannot have as much confidence in suggesting the direction of causation in the associations.

### Results

Supplemental Table 6 provides a description of the NS analytical sample on main demographics, income, and SES status, as well as clinical levels of mental health disorder at age 25.

#### GHQ-12 at Age 25

Shown in Supplemental Table 7 is the prevalence of probable mental health problems, as indicated by a GHQ-12 score ≥4, at age 25 in the NS sample. The overall prevalence was 25.3%, with little difference between participants assigned male and female at birth. It should be noted that Furukawa et al. (2003) found that Kessler 6 has better discriminatory power than GHQ-12 in detecting DSM-IV depressive and anxiety disorders, at least in large general population samples. The overall prevalence rates by the respective threshold scores are, therefore, unlikely to be directly comparable. Across the SES measures, there was a tendency for probable mental health problems to be more prevalent among the disadvantaged groups. We see a significantly higher occurrence in the lowest income quintile compared to the highest (*OR* = 1.26, *p* < .05); although, as in MCS, the pattern appears slightly non-monotonic, with the lowest rates found in the fourth income quintile, followed by the third quintile, and then the highest quintile. Reflecting the findings regarding subjective measures of SES in the MCS, the strongest inequality in mental health is seen for financial strain, with 59.6% of those finding it quite or very difficult having a GHQ-12 score of ≥4, compared to 13.2% of those who said they were living comfortably, an odds ratio difference of 9.70 (*p* < .001). In terms of NS-SEC, a difference was found between those in professional and managerial professions, compared to those in routine and semi-routine jobs (*OR* = 1.27, *p* < .05) and to those who were unclassifiable (*OR* = 2.78, *p* < .001). It is worth noting, however, that the lowest rates of mental health disorders were seen among those in the middle (NS-SEC 3 and 4). Those with no qualifications had a higher prevalence than those with the highest level of education (NVQ5) (*OR* = 1.43, *p* < .05). On both indices of SES disadvantage, we see that inequalities in mental health disorder rise for every additional disadvantage, with the strongest pattern found in Index A, which focuses on income-related variables.

#### Additional Analyses Examining Moderators

Additional analyses identified some potential moderators of the relationship between income/financial strain and mental health. Full tables in Excel format are available in the Supplemental Material, with potential moderators shown in Supplemental Tables 8.1 to 8.6. These show that the prevalence of psychiatric disorders varied on some moderating variables. Those living with parents or guardians had a higher prevalence (28.6%, 95% CIs [26.3, 30.9]) than those living independently (23.8%, CIs [22.3, 25.3]), whereas those living with a partner had a lower prevalence (20.4%, CIs [18.7, 22.3]) than those not living with a partner (28.6%, CIs [26.6, 30.3]). There was also a difference based on housing tenure, with those whose home was owned outright/with a mortgage having a lower prevalence of disorder (16.6%, CIs [17.2, 22.1]) compared to those renting/other (26.8%, CIs [25.3, 28.3]). However, in terms of the variables examined as moderation of the relationship between income/financial strain and mental health, the only fully significant moderation effect was for tenure, whereby the difference between those finding it difficult to manage financially compared to those living comfortably was much greater for those who owned their own homes (*OR* = 21.9) than those who did not own their own homes (*OR* = 8.4).

## Discussion

### Summary of Primary Findings

The results of this study indicate that the gradient between individual income and mental health at age 25 (in NS) and between household income and mental health at 14 and 17 (in MCS) are both relatively shallow and not fully monotonic. In the MCS, it is the second quintile that has a greater probability of clinical levels of mental health scores than the lowest, whereas in NS it is the highest quintile that has a greater probability than the third or fourth. Among parents or guardians of the 14- and 17-year-olds in the MCS, the gradient between household income and mental health is rather steep and fully monotonic, although it should be noted that these parents or guardians were overwhelmingly female (95%), compared with the other sample groups.

Related to this, inequalities in mental health problems between income groups are considerably smaller in adolescence and young adulthood than in adulthood. Significantly, financial strain (i.e., how well the parents or guardians of the 14- and 17-year-olds or participants themselves at age 25 feel they are managing financially) appears to be much more consistently and monotonically correlated with poor mental health across all groups, even when assessed by the parent or guardian of cohort members rather than the member themselves. Indices that add broader measures—such as financial strain, cohort member assessed wealth relative to friends, NS-SEC, and education—to income also display monotonic and often very steep, associations with mental health.

The MCS results support existing assumptions regarding relationships between reporting of mental illness and sex in under-18s, with female participants significantly more likely to report than male participants (McManus et al., 2016). However, by age 25 in NS, the difference by sex disappears. This may be explained in a number of different ways. Mental health problems may simply onset later for people assigned male, or cultural factors associated with masculinity may diminish self-reporting in childhood. Relatedly, we analyzed interactions between sex and our various markers of SES status and found no systematic patterns of interaction. There were signs of a steeper income gradient for female participants in the K6 measure of potential clinical depression at age 17, but this pattern was not found for other markers of SES status or the SMFQ measure at age 14.

### Strengths and Limitations of the Study

We used high-quality nationally representative survey data on the current generation of adolescents to provide quantitative estimates of risk differences relevant to UK policy to tackle the UK adolescent mental health crisis. We compared risk differences in early, mid, and late adolescence and provided extensive sensitivity analysis. While we cannot definitively rule out reverse causation bias, there are three considerations that may address concerns with our approach:

1. evidence already exists about causality (see “Work, income and the social determinants of ill-health”);
2. we are assuming only forward causation from parent or guardian income to adolescent mental health problems and not reverse causation from adolescent mental health problems to income, which is more plausible age 14 and 17 (where household income is used) but may be less plausible age 25 (where individual income is used); and
3. we believe that we present conservative underestimates because of relatively large measurement error in the adolescence of both mental health problems and income (see “Research implications,” below).

Beyond this, our analysis of three different mental health measures means that we cannot compare magnitudes between the different ages with complete confidence, while the income measure used at age 25 in NS is individual income and only from one wave of data, whereas the MCS measure was household income and averaged from age 9 months to 14 years. In addition, those analyzed in NS were born around 10 years before those in MCS, and the parents or guardians of MCS cohort members are split over a number of age groups, with the youngest being just 31 in wave 7 when their cohort member was 17. Finally, the parents or guardians of MCS cohort members are from a generation(s) even further removed from the 14- and 17-year-olds than are the NS cohort members, and the primary parents or guardians in our analysis are overwhelmingly (95%) female. There may be effects based on sex or gender that we do not analyze here. While recognizing these limitations, it is important to emphasize that the findings presented are produced using established and recognized methods and controls and are consistent with findings in other studies.

### Comparison With Other Studies

Some of the authors of this study have undertaken similar analysis (Parra-Mujica et al., 2023) of Understanding Society data covering 16- to 24-year-olds over 10 waves of that UK Household Longitudinal Survey. That study found a much more consistent monotonic association between household income and the SF-12 Mental Component Summary measure of mental health. This may be to do with the mental health measure used, which includes some physical symptoms in calculating the Mental Component Summary score (Ware et al., 1995), average household income having been derived from a larger number of more frequent waves of data, or more than one cohort having been included due to the structure of that survey. Importantly, though, gradients are present in both studies.

### Research Implications

We suspect that our finding regarding larger observed inequalities in adulthood than in adolescence is primarily due to larger measurement errors for both mental health problems and income during adolescence compared with adulthood. Mental health problems are measured with a relatively large error during adolescence because

1. it is hard to distinguish short-term adolescent mental health problems that are likely to resolve without long-term harm from long-term mental health problems that are likely to persist into adulthood; and
2. long-term mental health problems are under-reported by some adolescents (especially boys).

Income is measured with a relatively large error during adolescence. This is because adolescents receive substantial resource investments that do not show up in household income figures such as state-funded education and care services (e.g., informal gifts of time and money from extended family). As such, household income is a less accurate guide to material and social deprivation in adolescence than adulthood.

This measurement error may also play a role with regard to our findings on income versus other measures of SES status. This may explain why multiple markers of SES status may provide a more accurate estimate of the real level of material and social deprivation. It may also be partly to do with the close conceptual connection between parent or guardian subjective financial strain and parent or guardian subjective well-being, which means that the correlation between adolescent mental health problems and parent or guardian subjective financial difficulties may be vulnerable to reverse causation bias. In this case, adolescent mental health problems may cause parents or guardians subjective well-being problems including subjective financial difficulties.

Three other issues may play a role with regard to these findings. First, the shallow and non-monotonic (at the bottom two income quintiles for 14- and 17-year-olds) gradient in adolescence may not be a special feature of adolescence (e.g., to do with adolescent development or financial dependence) but instead an indication that the effects of low income on mental health are gradual, cumulative, and long term, taking decades to build to the full effect, and perhaps that these gradual effects become more powerful when they interact and combine with other strains in life such as having children. We cannot conclude this with a great degree of certainty, however, given that the primary parent or guardian sample is composed of 95% mothers and underlying drivers of the observed associations may exist based on sex. In addition, Patalay and Fitzsimons (2020) found variability in reporting of psychological distress at 17 in the MCS among individuals with lower family income. Compared to higher-income respondents, they were more likely to report high and low Kessler 6 scores (Patalay & Fitzsimons, 2020).

Second, other moderating variables may be present between income and mental health depending on age. In childhood and adolescence, some parents or guardians may be able to mitigate their children’s exposure to low income and inequalities and produce stable childhood environments at odds with the objective financial status of the family (see discussion of parenting in Pierron et al., 2018). This may explain why, at age 11, only 5% of MCS cohort members felt their families were poorer than those of their friends, although this could equally be due to a tendency to have friends with similar wealth. For parents or guardians, our finding that household income and financial strain are both similarly correlated with mental health may suggest, reasonably, that they are more directly exposed to the harms of low income.

Third, the features of financial strain that younger people are exposed to may not track net household income perfectly. It is possible that those in the second-lowest quintile may be exposed to greater day-to-day “low income,” perhaps as a result of their parents or guardians spending proportionally more than those from the lowest quintile on housing, for example, through having obtained a mortgage that proves difficult to repay. The findings from NS, in which the highest individual income quintile had a greater probability of clinical levels mental health scores than the third or fourth, may support this. High-income 25-year-olds, whose income is likely to be substantially below the highest quintile for older age groups, may have entered employment earlier and had sufficient income to secure a mortgage at a young age, but then find it difficult to repay due to high house prices or be left with less day-to-day income to deal with unpredictable events. They may live alone or with a partner and be unable to fall back on household income from others. This may be borne out in our analysis of moderating variables for this cohort. While living with a mortgage overall was associated with lower levels of mental health problems than renting, for those finding it difficult to manage financially, the opposite was true. These young people may be experiencing increased financial strain associated with loss of equity and the absence of alternatives. For those renting, lower levels of fear of destitution may exist by their not losing equity if they are unable to meet rent payments and also being more immediately eligible for housing support (see Smith et al., 2017). Rohe and Lindbland (2013) have identified a gradient within owner occupation stretching from security of outright ownership of residence to distress of repossession, with Cairney and Boyle (2004) distinguishing levels of distress between owners and those with mortgages.

Inequality may also play a role with regard to the second quintile “issue” in the MCS and the likelihood that subjective measures do not track income perfectly. Pickett and Wilkinson (2015), among others, have presented a range of epidemiological pathways for the impact of inequality on people’s health. Explanations for negative impact include the evolutionary concern of others having more resources by which to compete as well as social concerns regarding esteem. There is evidence of an impact of inequality on childhood cognitive functioning as a result of exposure to income-related long-term stress (Decker et al., 2020). Some of this study’s authors have argued elsewhere (M. T. Johnson & Johnson, 2019) that there is evidence of interpersonal domination inducing stress. In both explanations, stress is stimulated by exposure to extrinsic mortality cues, such as the threat of destitution. Higher levels of anxiety and depression among the second quintile than those above and below them may be because they are more likely to be exposed to broader points of SES comparison than those in the lowest quintile (see discussion of geographic inequality in Agrawal & Phillips, 2020). Unlike those in the lowest quintile, who may be more likely to be clustered in areas with those from similar SES backgrounds, those in the second may be more likely to share space with those from higher SES backgrounds, providing greater comparative sources of anxiety.

### Policy Implications

#### Universal Basic Income

These data provide qualification for Mayer’s claim that income is the “ultimate ‘multipurpose’ policy instrument” (Mayer, 1997, p. 145). An increase in net income overall need not necessarily mitigate mental health in all adolescents, although it appears that it would for the vast majority. However, there is *prima facie* reason to believe that an increase in income that people experience day-to-day may have an impact on all. Our 14 to 25-year-old cohort has suffered from the loss, in England at least, of the Educational Maintenance Allowance, which provided up to £30 per week to students who stayed in school between the ages of 16 and 19 and insisted that under-18s are expected to be dependent upon family or carers (Social Security Advisory Committee, 2018) at a time in which the cost-of-living crisis is placing increasing pressure on the ability of those adults to support themselves. Most forms of welfare are unavailable to under-18s, and the National Living Wage—which provides a higher statutory minimum than the National Minimum Wage—is only applicable from age 23 (Social Security Advisory Committee, 2018; UK Government, 2022). As such, UBI schemes designed for health impact by significantly increasing the income of lower quintiles and providing security and predictability of payment offer particular prospective benefits for young people (Chen et al., 2023). Indeed, Gibson et al.’s (2020, p. e173) systematic review of cash transfer programs that resemble UBI found modest to strong positive effects on health outcomes, including child mental health, with suggestions that underlying mechanisms could include “reduced stress, improved parenting quality, and reduced financial strain.”

#### Implications for Other Countries

There are likely to be a number of features of the UK context that are both comparable and distinct when seeking to extrapolate findings to other countries As a high-income country and advanced economy that has undergone a process of neoliberal economic reform since the 1980s, the UK has similarities to the United States, for example, that may enable the use of findings by US-based researchers and policymakers. In particular, the reduction in opportunities for young people to obtain secure income through employment that is sufficient to purchase a home and raise a family (Bialik & Fry, 2019), when compared with previous cohorts, is likely to lead to similar pressures on mental health in both countries. Also like the UK, the United States is experiencing increases in rates of anxiety and depression among children and young people (Lebrun-Harris et al., 2022). By contrast, while pressures on the NHS exist—particularly with regard to mental health services—in the wake of a decade of austerity, the COVID-19 pandemic, and the cost-of-living crisis, UK young people still have universal access to healthcare, which is free at the point of use. In the United States, lack of such access may result in worsening of mental health symptoms. In addition, financial implications could be even greater due to the relatively higher cost of healthcare (OECD, 2022). It is possible that factors such as these could result in even greater impact and cost savings from policies designed to increase income in the United States than in the UK.

## Conclusion

The data indicate strong associations between income and mental health. However, for younger people, associations may not always be straightforward. While the non-monotonic associations for 14- and 17-year-olds should not be overstated, subjective measures, or after housing costs income measures, might provide a better understanding of the financial circumstances young people experience. Given the disproportionate attrition rate among lower-SES groups and well-known and large measurement errors in both mental health problems and living standards during adolescence, it is possible that the associations we have described above are stronger within the population. This supports a *prima facie* case for redistributive policies that increase incomes progressively. With cash transfer trials planned or underway, this article provides additional guidance to policymakers in shaping those trials for impact on mental health among a vulnerable age cohort. There may be a need, in addition, to examine the issue of high-risk borrowing, which has an impact on disposable income and has been found elsewhere to be associated with distress (Wood et al., 2013). Large-scale experimental studies in a UK context are required to confirm our observational findings, with microsimulation to extrapolate results over the longer term likely to be of substantial benefit to researchers and policymakers.

## Supplemental Material

sj-xlsx-1-prv-10.1177_26320770231204993 – Supplemental material for How Far Can Interventions to Increase Income Improve Adolescent Mental Health? Evidence From the UK Millennium Cohort Study and Next Steps Supplemental material, sj-xlsx-1-prv-10.1177_26320770231204993 for How Far Can Interventions to Increase Income Improve Adolescent Mental Health? Evidence From the UK Millennium Cohort Study and Next Steps by Aase Villadsen, Elliott Aidan Johnson, Richard Cookson and Matthew Thomas Johnson in Journal of Prevention and Health Promotion
